# The Effect of Rosuvastatin on Inflammation, Matrix Turnover and Left Ventricular Remodeling in Dilated Cardiomyopathy: A Randomized, Controlled Trial

**DOI:** 10.1371/journal.pone.0089732

**Published:** 2014-02-25

**Authors:** Kaspar Broch, Erik T. Askevold, Erik Gjertsen, Thor Ueland, Arne Yndestad, Kristin Godang, Wenche Stueflotten, Johanna Andreassen, Rolf Svendsmark, Hans-Jørgen Smith, Svend Aakhus, Pål Aukrust, Lars Gullestad

**Affiliations:** 1 Department of Cardiology, Oslo University Hospital Rikshospitalet, Oslo, Norway; 2 Research Institute of Internal Medicine, Oslo University Hospital Rikshospitalet, Oslo, Norway; 3 Department of Endocrinology, Oslo University Hospital Rikshospitalet, Oslo, Norway; 4 Department of Radiology, Oslo University Hospital Rikshospitalet, Oslo, Norway; 5 Section of Clinical Immunology and Infectious Diseases, Oslo University Hospital Rikshospitalet, Oslo, Norway; 6 Department of Internal Medicine, Drammen Hospital, Vestre Viken Hospital Trust; Drammen, Norway; 7 Faculty of Medicine, University of Oslo, Oslo, Norway; 8 K. G. Jebsen Cardiac Research Centre and Center for Heart Failure Research, Faculty of Medicine, University of Oslo, Oslo, Norway; Texas A & M, Division of Cardiology, United States of America

## Abstract

**Background:**

Dilated cardiomyopathy is characterized by left ventricular dilatation and dysfunction. Inflammation and adverse remodeling of the extracellular matrix may be involved in the pathogenesis. Statins reduce levels of low density lipoprotein cholesterol, but may also attenuate inflammation and affect matrix remodeling. We hypothesized that treatment with rosuvastatin would reduce or even reverse left ventricular remodeling in dilated cardiomyopathy.

**Materials and Methods:**

In this multicenter, randomized, double blind, placebo-controlled study, 71 patients were randomized to 10 mg of rosuvastatin or matching placebo. Physical examination, blood sampling, echocardiography and cardiac magnetic resonance imaging were performed at baseline and at six months’ follow-up. The pre-specified primary end point was the change in left ventricular ejection fraction from baseline to six months.

**Results:**

Over all, left ventricular ejection fraction improved 5 percentage points over the duration of the study, but there was no difference in the change in left ventricular ejection fraction between patients allocated to rosuvastatin and those allocated to placebo. Whereas serum low density lipoprotein cholesterol concentration fell significantly in the treatment arm, rosuvastatin did not affect plasma or serum levels of a wide range of inflammatory variables, including C-reactive protein. The effect on markers of extracellular matrix remodeling was modest.

**Conclusion:**

Treatment with rosuvastatin does not improve left ventricular ejection fraction in patients with dilated cardiomyopathy.

**Trial Registration:**

ClinicalTrials.gov NCT00505154

## Introduction

Statins are inhibitors of 3-hydroxy 3-methylglutaryl coenzyme A reductase, the rate-limiting enzyme in cholesterol synthesis. Statins significantly reduce the risk of coronary events in patients with, or at risk of developing, coronary artery disease [1] [2]. While these effects are clearly related to lipid lowering, statins also possess anti-inflammatory and matrix stabilizing properties of potential relevance to their cardioprotective effects [3]. The relative risk reduction observed in statin trials is independent of baseline cholesterol levels [1] and is associated with a treatment-induced reduction of C-reactive protein (CRP) serum concentration [4].

The role of statins in heart failure (HF) has been under debate. Two major, randomized controlled trials in patients with HF, the CORONA [5] and GISSI-HF [6] trials, did not show any effect on survival with treatment with rosuvastatin, a statin that effectively reduces the number of coronary events in a low risk population [2]. However, in several small, randomized trials, treatment with a statin has been associated with improved left ventricular (LV) function and a reduction in markers of inflammation in patients with dilated cardiomyopathy (DCM) [7] [8] [9] [10]. A meta-analysis, including the CORONA and GISSI trials, recently concluded that there is evidence to suggest that in patients with HF, statins may improve LV ejection fraction (LVEF) and reduce the number of hospitalizations for worsening HF [11]. It is unclear, however, if these effects are related to changes in immunological parameters or remodeling of the extracellular matrix.

Approximately 10% of HF cases are due to DCM [12]. Dilated cardiomyopathy is a clinical entity based on morphological criteria and exclusion of specific causes of LV dysfunction such as coronary artery disease, valvular disease or hypertension [13]. This phenotype is probably the result of a multifactorial process where external or internal stressors induce structural changes in the myocardium of individuals at risk. Dilated cardiomyopathy may thus serve as a model for studying pathogenic mechanisms in the failing myocardium *per se,* without one having to account for the confounding effect of coronary atherosclerosis. In the present study, we evaluated the effect of rosuvastatin in patients with idiopathic DCM, hypothesizing that rosuvastatin would reduce or even reverse LV remodeling in this patient population through effects on inflammation and matrix remodeling.

## Materials and Methods

### Study Procedures

This is a multicenter, randomized, double blind, placebo-controlled trial designed to assess the effect of rosuvastatin on LV function in patients with DCM (ClinicalTrials.gov, registration number NCT00505154; http://clinicaltrials.gov/ct2/show/NCT00505154). It was conducted at three sites in Norway. The investigation conforms with the principles outlined in the Declaration of Helsinki and was approved by the Regional Committee for Medical and Health Research Ethics South East and the Norwegian Medicines Agency. All patients provided written, informed consent. The study was performed in accordance with the Consolidated Standards of Reporting Trials (CONSORT) statement [14]. The protocol for this trial and supporting CONSORT checklist are available as supporting information; see Protocol S1 and Checklist S1.

At baseline, all participants underwent physical examination, blood tests, echocardiography and, unless contraindicated, cardiac magnetic resonance imaging (MRI). Patients were then randomized to 10 mg of rosuvastatin or matching placebo, once daily, in a 1:1 fashion. After four weeks and three months, the patients were reassessed for safety. Physical examination, blood tests, echocardiography and cardiac MRI were repeated after six months of intervention.

### Patient population

Patients between 18 and 80 years old with symptoms and/or signs of HF for at least three months and with LVEF ≤ 40% were eligible, provided that treatment with a cholesterol-lowering drug was not otherwise indicated. Criteria for exclusion included decompensated HF requiring mechanical or inotropic support; HF of ischemic etiology; hemodynamically significant valvular disease not considered to be secondary to ventricular dilatation; recent or planned surgical procedures or operations; significant concomitant disease such as infection, severe pulmonary disease or connective tissue disease; acute or chronic liver disease; current statin treatment; or contraindications to statin therapy defined as hypersensitivity to any statin, alanine transaminase ≥ 2 times the upper limit of normal, serum creatinine ≥ 2 mg/dL, an unexplained creatine kinase ≥ 3 times the upper limit of normal, current or planned pregnancy, or breast feeding.

### Drug handling, randomization and blinding

Rosuvastatin and matching placebo tablets were provided by the manufacturer (AstraZeneca) and appropriately stored. A randomization list was produced by an external department by computer block randomization and placed in a concealed envelope. Study drugs were provided in numbered, otherwise indistinguishable containers and dispensed in a double blind fashion by a dedicated study nurse. Study drug adherence was evaluated at one, three and six months’ follow-up based on pill counts of returned, unused study medication. Compliance was considered good if more than 80% of the appropriate number of tablets had been taken.

### Study outcomes

The primary objective of this study was to evaluate the effect of rosuvastatin on LV remodeling in patients with HF due to idiopathic DCM. The pre-specified primary end point was the change in LVEF from baseline to follow-up as analyzed by MRI. Due to the fact that many otherwise eligible patients had contraindications to cardiac MRI, we chose to report the change in LVEF as determined by MRI or, if the latter result was not available at both baseline and follow-up, by echocardiography. Secondary endpoints were effects on neurohormonal variables, immunological variables and markers of extracellular matrix remodeling; LV end-diastolic and end-systolic volumes as measured by MRI and echocardiography; New York Heart Association (NYHA) functional class; and quality of life. Withdrawals and side effects were recorded.

### Imaging

MRI and echocardiography were performed at baseline, before study drug administration, and after six months, prior to study drug discontinuation. All image analyses were performed at Oslo University Hospital, Rikshospitalet, which served as the core laboratory. Image analyses were performed by operators blinded to patient treatment allocation.

### Magnetic resonance imaging

Siemens 1.5 tesla scanners were used for MRI (Siemens Avanto and Siemens Sonata; Siemens Medical Systems, Erlangen, Germany). LV long axis and short axis views were acquired using a breath-hold, prospectively ECG-triggered, segmented, balanced, steady-state free precession gradient-echo cine sequence with minimum echo and repetition times, 6 mm slice thickness, 4 mm short-axis interslice gap, spatial resolution 1.9 mm×1.3 mm, and temporal resolution 30–35ms. LV endocardial borders were traced manually using a PACS work station (Sectra Medical Systems AB, Linköping, Sweden), and LV volumes and ejection fractions were calculated by short axis slice summation.

### Echocardiography

Echocardiography was performed with Vivid 7 or E9 ultrasound scanners (GE Vingmed Ultrasound, Horten, Norway), using phased array transducers. Cine loops were digitally stored and later analyzed off line using Echo-Pac (GE Vingmed). 2D parameters and conventional Doppler parameters were measured according to current recommendations [15] [16]. Valvular regurgitations were graded as mild, moderate or severe by visual assessment [17]. LVEF was measured by Simpson’s biplane method [15].

### Blood sampling

Peripheral blood was collected in pyrogen-free tubes without any additives (serum) or with EDTA as anticoagulant (plasma). The tubes were allowed to coagulate at room temperature (serum) or put on melting ice (plasma) and centrifuged after coagulation (1000*g* at 10 minutes) or within 10 minutes (2000 *g* at 20 minutes) to obtain serum and platelet-poor plasma, respectively. Serum and plasma samples were stored at –80°C in multiple aliquots and thawed once only.

### Biochemical analyses

N-terminal pro-B-type natriuretic peptide (NT-proBNP) was analyzed by routine methods on a MODULAR analytical platform by an electrochemiluminescence immunoassay (Roche proBNP II, Roche Diagnostic, Basel, Switzerland). Levels of CRP were determined on a MODULAR Analytical platform, P800 module (Roche Diagnostics) using a particle-enhanced immunoturbidimetric assay (Tina-Quant CRP Gen.3). Soluble tumor necrosis factor receptor type 1 (sTNF-R1), osteoprotegerin (OPG), soluble glycoprotein 130 (sgp130), matrix metalloproteinase-9 (MMP-9) and monocyte chemotactic protein-1 (MCP-1)/CCL2 were analyzed by enzyme immunoassays obtained from R&D Systems (Minneapolis, MN).

Procollagen type I and III N-terminal pro-peptides (PINP and PIIINP, respectively) were analyzed by radioimmunoassays (UniQ PINP RIA and UniQ PIIINP RIA, Orion Diagnostica, Espoo, Finland). Von Willebrand factor (vWF) was determined by an enzyme immunoassay as previously reported [18]. Inter-and intra-assay coefficients of variation were < 10% for all biochemical analyses.

### Quality of life assessment

Quality of life was assessed at baseline and after six months using self-report inventories. Heart failure related symptoms were measured with the Minnesota Living with Heart Failure Questionnaire (MLHFQ) and the EuroQol visual analogue scale. Symptoms of depression were assessed using the Beck Depression Inventory.

### Statistics

Assuming a standard deviation of 7.5% in LVEF measurement, we calculated that with an α of 5% and power of 80% we would need 32 patients in each group in order to observe a 5 per cent points difference in the change in LVEF between the rosuvastatin and placebo arms at follow-up. To allow for drop out, we aimed to enroll 75 patients.

To assess total population differences between baseline and follow-up, we used paired parametric (t-tests) or non-parametric (Wilcoxon signed rank) tests depending on distribution. Differences in numerical outcome variables between patients treated with rosuvastatin and patients treated with placebo were analyzed using analysis of covariance (ANCOVA), adjusting for baseline values [19]. Highly skewed parameters were log transformed prior to analysis. The number of adverse events was compared across treatment groups by Poisson regression. The between-group difference in the change in NYHA class from baseline to follow-up was analyzed by general estimating equations. All end point analyses were performed according to the intention-to-treat principle. Numerical values are presented as mean values ± SD or median (interquartile range) as appropriate. All statistical analyses were performed with the Statistical Package for Social Sciences version 18 software (SPSS Inc. Chicago, IL). Two-sided probability values were considered significant at p < 0.05.

## Results

### Patients

**Figure 1:**
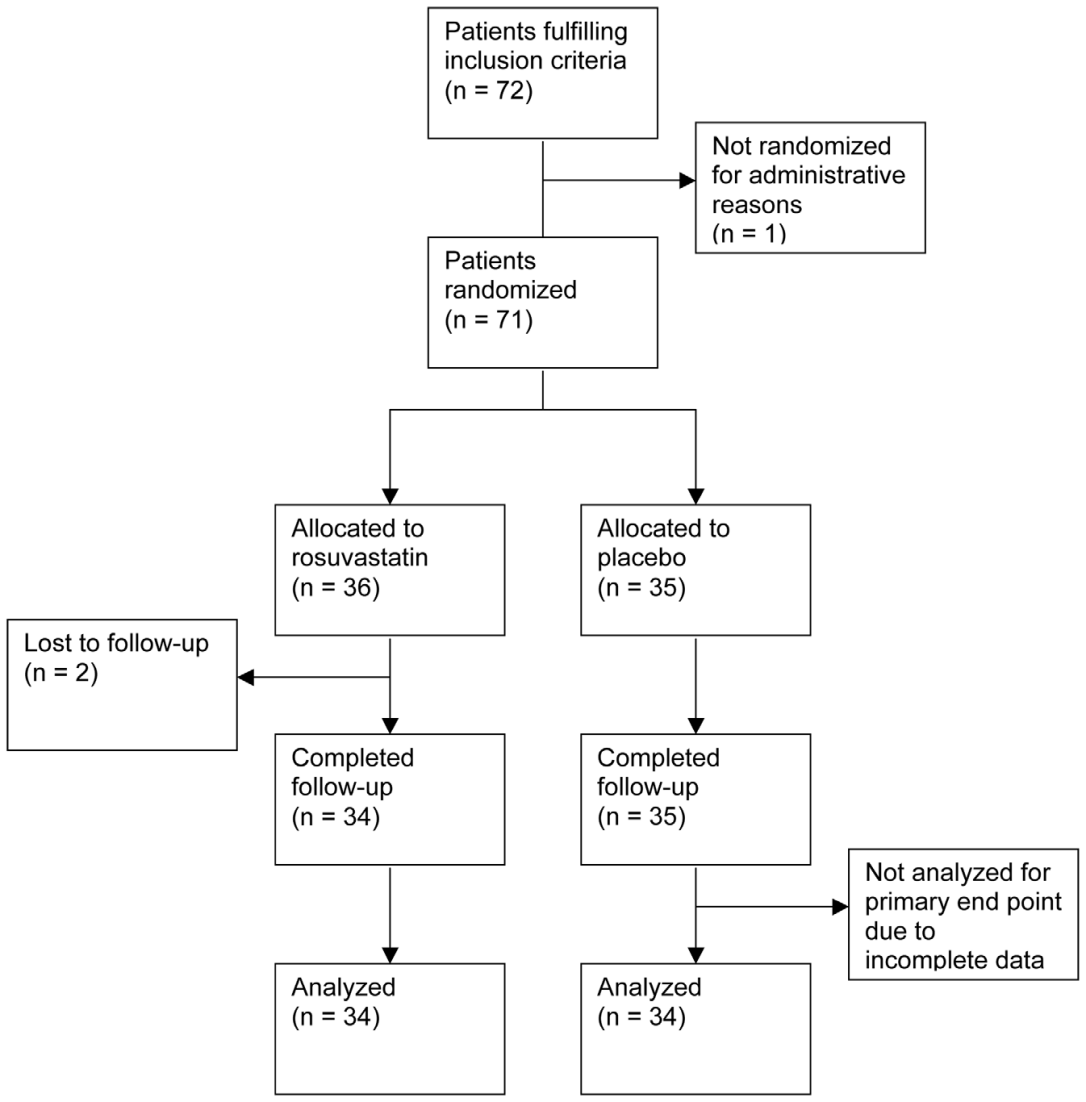
Trial flow chart.

From the 20^th^ of September 2007 to the 15^th^ of February 2012, a total of 72 patients were included in Oslo (n  =  57), Drammen (n  =  12) and Trondheim (n  =  3). A DCM phenotype had been confirmed by echocardiography prior to inclusion. Ischemic etiology had been excluded by a combination of patient history, coronary angiography (n  =  71) and/or myocardial scintigraphy. Primary valvular disease was excluded by a combination of patient history and echocardiography. For administrative reasons, one patient was never randomized and was excluded from further participation in the study. Seventy-one patients were thus randomized, of whom 36 were allocated to treatment with rosuvastatin and 35 to placebo. Baseline characteristics stratified by treatment allocation are presented in Table 1. Sixty seven patients were naïve to statins, whereas five patients had stopped taking statins 5 – 61 months prior to inclusion. Throughout the study period, heart failure treatment was adjusted in an individualized fashion according to current guidelines [20]. One patient discontinued study drug treatment after 5 days and was later lost to follow up. One patient withdrew consent without stating any particular reason. Both of these patients were in the rosuvastatin arm. A total of 69 patients were thus re-evaluated after 182±20 days. In one patient, imaging data were incomplete, and this patient was excluded from analysis of the primary endpoint (Figure 1).

**Table 1:** Baseline data stratified according to treatment allocation.

Variable	All patients	Rosuvastatin (N = 36)	Placebo (N = 35)
*Clinical characteristics*			
Age - years	58±11	58±11	58±11
Men – no (%)	55 (78)	27 (75)	28 (80)
Body mass index	28±5	28±5	28±5
Systolic blood pressure - mm Hg	125±20	128±23	123±17
Diastolic blood pressure - mm Hg	77±10	77±11	76±9
Heart rate – beats/minute	67±12	66±11	69±13
Atrial fibrillation – no (%)	15 (21)	3 (8)	12 (34)
NYHA class I – no (%)	8 (11)	4 (11)	4 (11)
NYHA class II – no (%)	43 (62)	21 (58)	22 (63)
NYHA class III – no (%)	20 (28)	11 (31)	9 (26)
*Medical history*			
Smokers – no (%)	13 (18)	6 (17)	7 (20)
History of hypertension – no (%)	21 (30)	17 (47)	4 (11)
Diabetes mellitus – no (%)	10 (14)	7 (19)	3 (7)
Prior stroke/TIA – no (%)	2 (3)	1 (3)	1 (3)
*Baseline medication*			
ACEI and/or ARB no (%)	71 (100)	36 (100)	35 (100)
Beta blocker – no (%)	70 (99)	35 (97)	35 (100)
Aldosterone antagonist – no (%)	30 (42)	18 (50)	12 (34)
Diuretic – no (%)	49 (69)	26 (72)	23 (66)
Digoxin or digitoxin – no (%)	13 (18)	5 (14)	8 (23)
*Laboratory values*			
Creatinine (mmol/l) [mg/dl]	85±19 [0.96±0.21]	86±18 [0.97±0.21]	84±20.[0.95±0.22]
Total serum cholesterol – mmol/l	5.8±1.2	5.9±1.6	5.6±0.8
C-reactive protein – mg/l	2.2 (1.0–5.3)	1.7 (1.0–1.4)	2.7 (1.3–6.2)
N-terminal pro-B-type natriuretic peptide – pg/dl	592 (236–1607)	600 (186–1630)	588 (243–1643)

NYHA: New York Heart Association; TIA: transient ischemic attack; ACEI: angiotensin converting enzyme inhibitor; ARB: angiotensin receptor blocker.

### Left ventricular remodeling and function

Magnetic resonance imaging was performed in 53 patients at baseline. Thirteen patients had an implantable device prohibiting cardiac MRI, in one patient MRI was contraindicated due to anxiety, and in three patients, MRI was not performed for administrative reasons. Fifty of the patients in whom MRI was performed at baseline, also had an MRI at six months’ follow-up. Echocardiographic images were available for all participants who were not lost to follow up, but in four patients, echocardiographic image quality did not allow for quantification of LVEF. In 68 patients, 34 in the rosuvastatin group and 34 in the placebo group, LVEF was available by MRI and/or echocardiography at baseline as well as follow-up.

**Figure 2:**
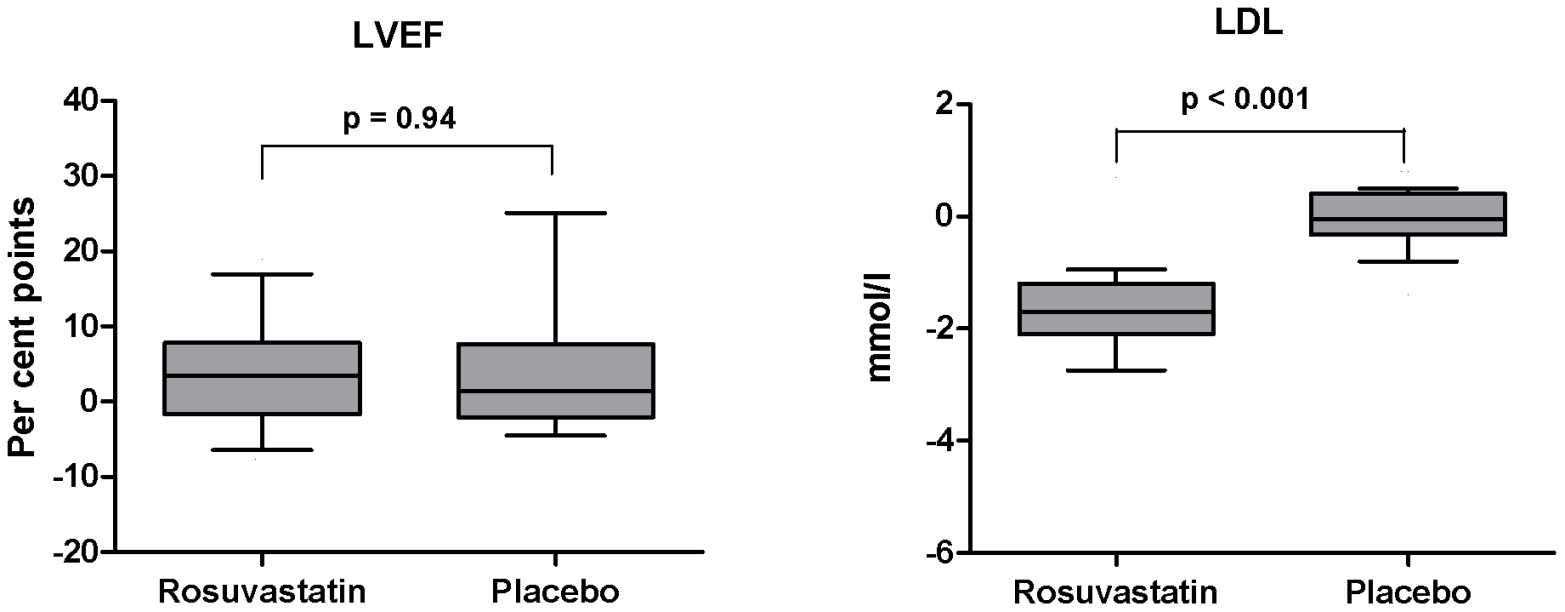
Changes in left ventricular ejection fraction and LDL cholesterol. The left panel illustrates the change in left ventricular ejection fraction (LVEF) stratified by treatment allocation. There was no difference in the change in LVEF between the two groups as analyzed by an independent group t-test (p  =  0.94). The right panel illustrates the change in LDL cholesterol stratified by treatment allocation. The fall in LDL cholesterol occurred in patients allocated to rosuvastatin (p for difference < 0.001). Boxes: 25–75 percentiles; whiskers: 10–90 percentiles.

**Figure 3:**
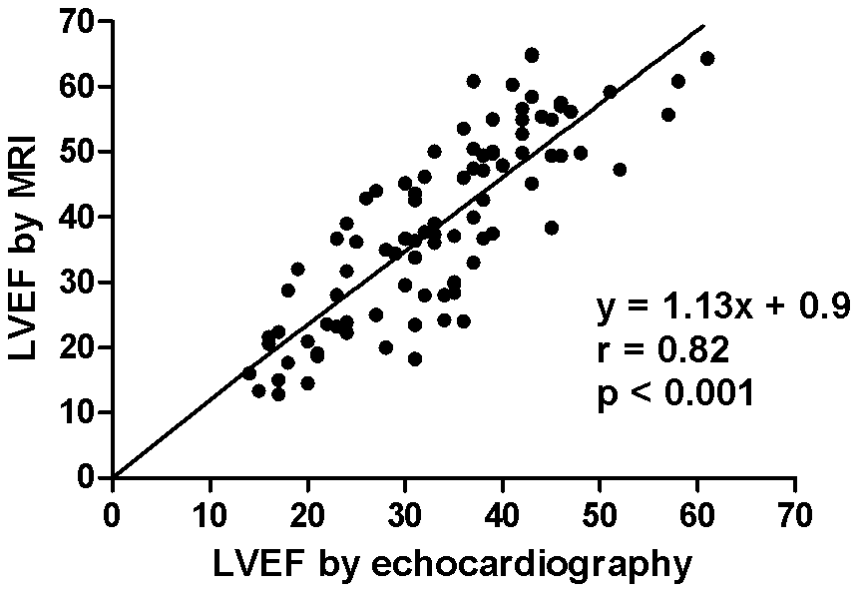
Correlation between left ventricular ejection fraction as measured by echocardiography and as measured by magnetic resonance imaging. Data from baseline and follow-up are pooled. The Pearson correlation coefficient between results obtained by magnetic resonance imaging (MRI) and results obtained by echocardiography was 0.82 (p < 0.001).

Over all, LVEF improved 4.6 percentage points over the duration of the study (95% confidence interval 2.2–7.0; p < 0.001), but there was no difference in the adjusted LVEF between the two treatment groups after six months (Figure 2 and Table 2). The two treatment groups were well balanced for baseline characteristics. However, there was a four times higher proportion of previously hypertensive patients in the rosuvastatin arm, and a four times higher proportion of patients with atrial fibrillation in the placebo arm. Adjusting for these differences did not affect the main result. A similar pattern as for LVEF was seen for LV end diastolic and end systolic size; despite a reduction in volumes across both treatment arms, there was no between-group difference in the outcome (Table 2). Results obtained by cardiac MRI and echocardiography were highly congruous (Figure 3).

**Table 2:** Results from cardiac imaging exams, New York Heart Association (NYHA) classification and quality of life measurements.

Variable	Placebo			Rosuvastatin			p-value for between-group difference
	Baseline	Follow-up	Change (95% CI)	Baseline	Follow-up	Change (95% CI)	
*Imaging*							
LVEF (%)*	36±13	40±14	4.7 (0.9 – 8.5)^†^	36±13	41±15	4.5 (1.5 – 7.5)^†^	0.98
EDV by MRI (ml)	249 (185 – 325)	202 (160 – 303)	–36 (–61 – –10)^†^	242 (191 – 326)	193 (169 – 326)	–26 (–48 – –3)^†^	0.56
ESV by MRI (ml)	159 (93 – 242)	118 (79–216)	–35 (–60 – –9)^†^	154 (92 – 236)	102 (67 – 176)	–28 (–49 – –7)^†^	0.76
LVEF by MRI (%)	36±14	41±15	5.4 (0.9 – 9.9)^†^	38±14	44±15	5.8 (2.0 – 9.5)^†^	0.75
EDV by E/D (ml)	188 (141 – 228)	151 (134 – 210)	–19 (–32 – –7)^†^	202 (145 – 278)	186 (138 – 270)	–2 (−17 – 13)	0.07
ESV by E/D (ml)	129 (87 – 165)	95 (79 – 148)	–19 (–32 – –6)^†^	135 (99 – 203)	126 (78 – 204)	–6 (−20 – 7)	0.16
LVEF by E/D (%)	32±10	36±10	3.9 (1.0 – 6.6)^†^	31±8	34±11	3.7 (0.9 – 6.6)^†^	0.81
*NYHA class*							
NYHA class (I/II/III)	4/22/9	7/22/6		4/21/11	5/26/3		0.90
*Quality of life*							
MLHFQ	23 (4 – 35)	14 (3 – 23)	–3 (−9 – 2)	30 (16 – 56)	20 (9 – 44)	–7 (−12 – –1)^†^	0.39
Beck depression inventory	4 (2 – 7)	3 (1 – 5)	–1 (−2 – 0)	6 (2 – 9)	3 (1 – 9)	–1 (−3 – 0)	0.91
EuroQoL VAS (%)	66±17	68±15	2 (−4 – 7)	64±18	66±18	1 (−7 – 8)	0.68

*This row represents left ventricular ejection fraction (LVEF) by magnetic resonance imaging (MRI) or, if the latter result was not available at both baseline and follow-up, LVEF by echocardiography (E/D, N = 18). ^†^ p < 0.05 for difference between baseline and follow-up. CI: confidence interval; EDV: end diastolic left ventricular internal volume; ESV: end systolic left ventricular internal volume; NT-proBNP: N-terminal pro-B-type natriuretic peptide; LDL: low density lipoprotein; MLHFQ: Minnesota Living with Heart Failure Questionnaire; VAS: visual analogue scale.

### Lipids and markers of inflammation, endothelial reactivity and extracellular matrix turnover

**Figure 4:**
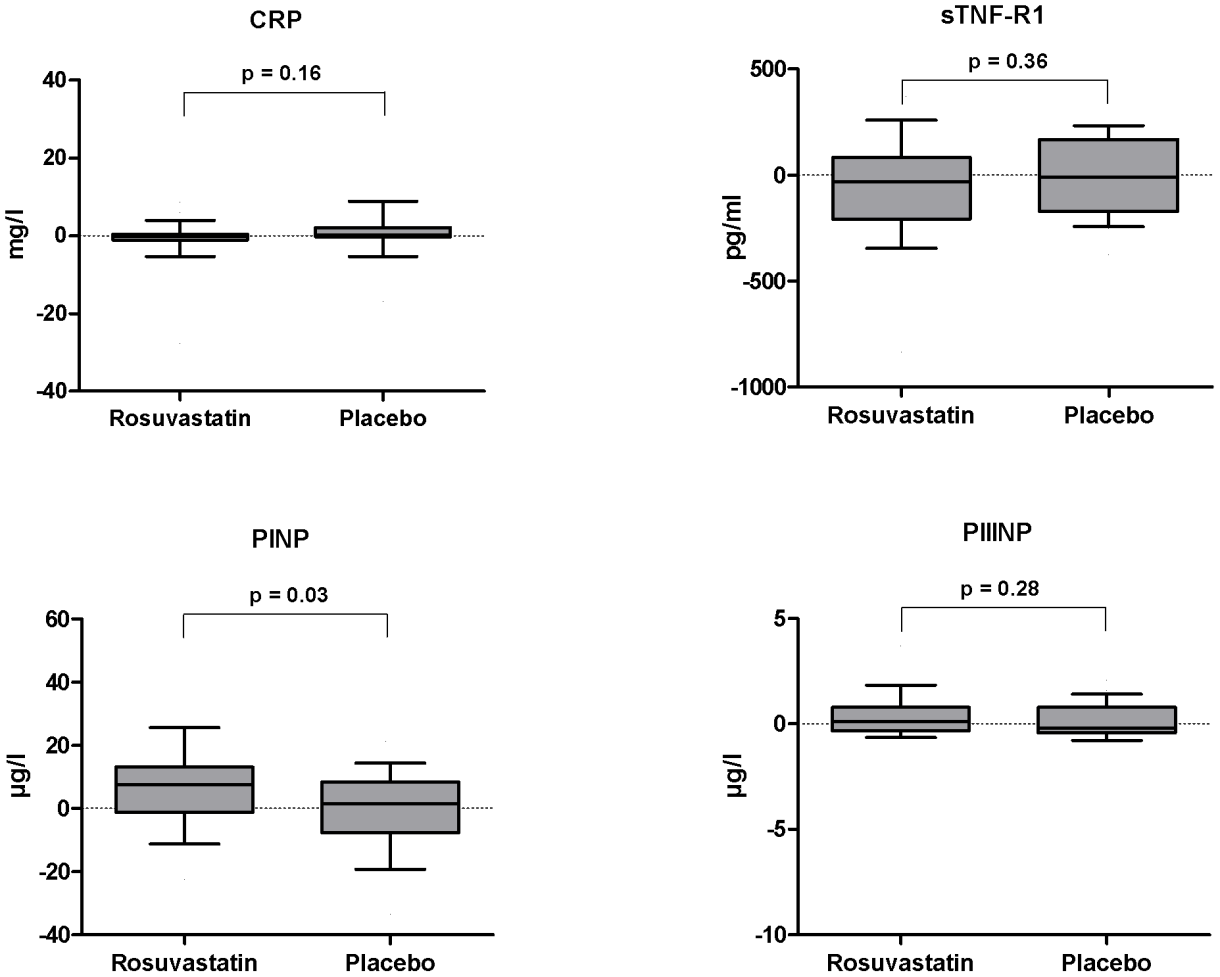
Changes in markers of inflammation and extracellular matrix turnover. Changes in C-reactive protein (CRP), soluble tumor necrosis factor receptor type 1 (sTNF-R1), and procollagen type I and III N-terminal pro-peptides (PINP and PIIINP) stratified by treatment allocation. p-values for between-group differences in changes were computed by independent group t-tests. While PINP increased more in patients treated with rosuvastatin as compared to patients treated with placebo (p  =  0.03), treatment did not affect any of the other markers of inflammation or matrix remodeling. Boxes: 25–75 percentiles; whiskers: 5–95 percentiles.

Laboratory results are presented in Table 3. Consistent with a high level of adherence to the study drug regimen, a substantial reduction in total and LDL cholesterol was observed in patients treated with rosuvastatin, resulting in a highly significant between-group difference (Figure 2 and table 3). In contrast, markers of inflammation, i.e. CRP, sTNF-R1, MCP1, sgp130 and OPG [21] and endothelial reactivity (vWF) [22] remained essentially unchanged throughout the study, and no difference was observed between treatment groups (Table 3 and Figure 4).

**Table 3:** Results of blood tests.

Variable	Placebo			Rosuvastatin			p-value for between- group difference
	Baseline	Follow-up	Change (95% CI)	Baseline	Follow-up	Change (95% CI)	
*Lipids*							
Cholesterol (mmol/l)	5.6±0.8	5.6±0.8	−0.1 (−0.3 – 0.2)	5.9±1.6	4.3±1.0	−1.7 (−2.1 − −1.3)*	<0.001
LDL cholesterol (mmol/l)	3.7±0.8	3.6±0.9	−0.1 (−0.2 − 0.1)	3.8±1.2	2.1±0.8	−1.7 (−2.0 − −1.4)*	<0.001
HDL cholesterol (mmol/l)	1.2±0.3	1.2±0.4	0 (−0.1 − 0.1)	1.3±0.4	1.4±0.5	0.1 (0 − 0.2)	0.19
*Natriuretic peptides*							
NT-ProBNP (pg/dl)	617 (245 − 1581)	499 (167 − 1226)	−79 (−347 − 307)	567 (186 − 1615)	431 (169 − 983)	−19 (−418 − 475)	0.73
*Kidney function*							
Creatinine (µmol/l) [mg/dl]	84±20	82±14	−2 (−6 − 3)	86±18	86±18	1 (−2 − 3)	0.23
*Markers of inflammation*							
C−reactive protein (mg/l)	2.7 (1.3 − 6.2)	3.2 (1.2 − 10.1)	1.1 (−1.3 − 3.5)	1.7 (1.0 − 4.0)	2.1 (1.0 − 4.9)	1.2 (−1.1 − 3.4)	0.14
sTNF-R1 (pg/ml)	1039±271	1038 ± 304	−1 (−71 – 68)	1134±327	1082 ± 235	−53 (−141 – 36)	0.72
MCP-1 (pg/ml)	115 (72 – 160)	117 (76 – 142)	−5 (−21 – 10)	103 (87 – 146)	94 (70−127)	−6 (−21 – 8)	0.77
gp130 (ng/ml)	92±23	96 ± 23	4 (−5 – 14)	91±18	97±22	6 (−3 – 15)	0.83
Osteoprotegerin (pg/ml)	666±243	610 ± 193	−56 (−127 – 16)	600±203	643±248	42 (−34 – 118)	0.13
*Endothelial reactivity*							
vWF (ng/ml)	21±14	22 ± 13	1 (−3 – 5)	23±16	23 ± 12	0 (−4 – 4)	0.99
*Matrix turnover*							
MMP-9 (ng/ml)	247 (50 – 386)	354 (162 – 467)	68 (11 – 124)*	220 (64 – 302)	243 (106 – 367)	13 (−33 – 59)	0.08
PINP (µg/l)	40 (29 – 47)	40 (27 – 50)	0 (−5 – 4)	38 (32 – 46)	45 (37 – 68)	8 (2 – 13)*	0.02
PIIINP (µg/l)	3.7 (3.3 – 4.4)	3.8 (3.4 – 5.0)	0.0 (−0.6 – 0.5)	3.8 (2.9 – 4.5)	3.8 (3.4−5.0)	−0.2 (−0.4 – 0.8)	0.27

* p < 0.05 for difference between baseline and follow-up. CI: confidence interval; LDL: low density lipoprotein; HDL: high density lipoprotein; NT-proBNP: N-terminal pro-B-type natriuretic peptide; sTNF-R1: soluble tumour necrosis factor receptor 1; MCP-1: monocyte chemotactic protein-1; gp130: Soluble glycoprotein 130; MMP-9: matrix metalloproteinase-9; PINP: procollagen type I N-terminal pro-peptide; PIIINP: procollagen type III N-terminal pro-peptide.

Among the candidate markers of extracellular matrix remodeling: MMP-9, PINP and PIIINP [23] [24] [25]; PINP and MMP-9 increased significantly throughout the study period for the population as a whole (p  =  0.03 and 0.03, respectively). Procollagen type I N-terminal pro-peptide increased more in patients treated with rosuvastatin as compared with patients treated with placebo (Table 3 and Figure 4), while for MMP-9 there was a trend toward a more pronounced increase in the placebo group (p  =  0.08) (Table 3).

### New York Heart Association class and quality of life

At baseline, 8/43/20 patients were in NYHA class I/II/III, respectively. In the 69 patients who completed the study, the corresponding figures were 12/48/9 at follow-up, indicating a significant improvement (p  =  0.003). However, no difference was observed between patients treated with rosuvastatin and those treated with placebo (p  =  0.90). Likewise, quality of life improved significantly in the population as a whole, but adjusted for baseline values, there was no between-group difference at follow-up (Table 2).

### Compliance, safety, and side effects

In the rosuvastatin arm, two patients, one of whom was later lost to follow-up, prematurely discontinued study drug due to mild side-effects. One patient in the placebo arm stopped taking the study drug after a few days due to stomach pain and diarrhea. Throughout the study period, a total of fifty adverse clinical events were recorded in 33 patients. Twenty-six events were recorded in 16 patients who were allocated to rosuvastatin, and 24 events were recorded in 17 patients allocated to placebo (p  =  0.80). Four serious adverse events were recorded in four patients allocated to rosuvastatin (surgery for colon cancer; development of atrial fibrillation; hospitalization for worsening HF; and an accidental fracture), and seven serious adverse events occurred in five patients allocated to placebo (development of dementia; and hospitalizations for hypotension, pneumonia, newly developed symptomatic coronary artery disease, and severe nose bleed). There were no episodes of rhabdomyolysis or on-treatment elevation in alanine transaminase. No patient died during follow-up.

## Discussion

Assuming that statins modulate inflammation and the extracellular matrix, we hypothesized that treatment with rosuvastatin would mitigate LV remodeling in patients with DCM. However, we did not find that six months of treatment with this potent statin influenced any measure of LV function or size in our patients. Moreover, while treatment with rosuvastatin was associated with a pronounced reduction in serum cholesterol, it did not affect plasma/serum levels of a wide range of inflammatory variables. The effect on markers of extracellular matrix turnover was modest.

There is a large body of evidence indicating that inflammation is involved in the pathogenesis of HF [26]. The anti-inflammatory effect of statins has also been extensively studied [27]. Almost uniformly, an on-treatment fall in CRP has been observed in major statin trials, most of which have been performed in patients with, or at increased risk of, atherosclerotic disease [2] [28] [29]. We have previously demonstrated that the anti-inflammatory effects of these drugs extend beyond a reduction in CRP [30] [31]. However, in the present study, rosuvastatin had no effect on markers of inflammation, including CRP.

The precise mechanisms underlying the proposed anti-inflammatory effect of statins have not been fully elucidated. The so called pleiotropic effects of statins could at least partly involve reduced inflammation secondary to a reduction in LDL levels [32]. In our patients, however, we observed a substantial on-treatment decrease in LDL, without this translating to an effect on inflammatory markers. It is possible that the anti-inflammatory effects of statins observed in atherosclerotic disease can be directly attributed to an amelioration of the atherosclerotic burden. However, in a large population of elderly patients with HF of ischemic etiology, rosuvastatin reduced levels of CRP [33], but its effect on other inflammatory markers has been disappointing [34] [35] [36]. Although the exact mechanism is not clear, it is therefore possible that the ability of statins to modulate inflammation in HF is questionable.

In the UNIVERSE trial [37], assessing the effect of rosuvastatin in DCM, an increase in PIIINP was observed in the rosuvastatin group. The authors speculated that an on-treatment increase in extracellular fibrosis might offset other, potentially beneficial effects of rosuvastatin, explaining the neutral result on LVEF in this study [38]. In the present trial, rosuvastatin was associated with an increase in PINP and an attenuated increase in MMP-9, potentially reflecting increased collagen synthesis and inhibited matrix degradation, respectively. While these processes could influence myocardial remodeling, the between-group differences were modest and, in the case of MMP-9, not significant. Most importantly, they were not accompanied by a treatment induced effect on LV function.

In the population as a whole, we observed a substantial increase in LVEF from baseline to follow-up. Implicitly, our study demonstrates the inherent potential for improvement in patients with DCM, and that adverse remodeling is not an irreversible process. The high degree of spontaneous recovery may have influenced our results. If new treatments for DCM are to prove better than placebo, they will have to not only attenuate adverse remodeling, but also contribute to a marked improvement in LV function.

### Limitations

The present study was powered to detect a five per cent point difference in LVEF between the two treatment arms. It could be argued that this endpoint was overly ambitious, necessitating a neutral outcome of the trial. On the other hand, we did not observe differences across treatment arms on any measure of LV size or function, including NT-pro-BNP, indicating that rosuvastatin did in fact not impact LV remodeling. Nonetheless, our study was underpowered to detect small, but potentially relevant changes in LV function. Due to the limited number of patients and short observation period, we cannot conclude that statin treatment does not reduce morbidity or mortality in patients with DCM.

“Idiopathic” DCM is a category that includes different disease entities with a common structural phenotype. While subgroups of these patients might benefit from statin treatment, others may not. This fact may limit the generalizability of our findings.

Finally, unlike most statins, rosuvastatin is a hydrophilic compound. Accordingly, it has been suggested that it does not have the same pleiotropic profile as the lipid soluble statins [11], and we cannot exclude that other statins might have performed differently in our patient population. In patients at risk of coronary disease, however, rosuvastatin is highly effective not only when it comes to reducing LDL cholesterol; it also effectively reduces CRP, as well as the number of cardiovascular events and death [2].

## Conclusion

This small, randomized study did not provide evidence that treatment with a potent statin improves LV function in patients with DCM. While safe, rosuvastatin should not be prescribed routinely in patients with HF. Whether treatment with a statin might benefit subgroups of patients with DCM, remains to be elucidated.

## Supporting Information

Checklist S1 **Consolidated Standards of Reporting Trials (CONSORT) checklist to show that the trial conforms to the CONSORT requirements.**
(DOC)

Protocol S1 **The full version of the trial protocol.**
(DOC)
