# Supplementary material for: The Effect of Rosuvastatin on Inflammation, Matrix Turnover and Left Ventricular Remodeling in Dilated Cardiomyopathy: A Randomized, Controlled Trial
Source: PLoS One. 2014 Feb 25;9(2):e89732. doi: 10.1371/journal.pone.0089732 (PMC3934914; doi:10.1371/journal.pone.0089732)
Supplement: Protocol S1 — The full version of the trial protocol. (DOC) [file pone.0089732.s002.doc]

Effect of Rosuvastatin on left ventricular remodeling and inflammatory markers in idiopathic dilated cardiomyopathy

EUDRACTNR 2007-000018-36.

Lars Gullestad, MD, PhD; John Kjekshus, MD, PhD; Thor Edvardsen, MD, PhD; Christen Dahl, MD; Svend Aakhus, MD,PhD; Erik Askevold, MD, Kaspar Broch, MD, Rita Skårdal, Hans Jørgen Smith, MD,PhD; Thor Ueland, PhD; Arne Yndestad, MSc; Pål Aukrust, MD, PhD;

*Rikshospitalet, University of Oslo, N-0027 Oslo, Norway*

Rune Mo MD, PhD: *Department of Cardiology, St, Olavs Hospital, Trondheim*

Erik Gjertsen MD, *Department of Internal Medicine, Buskerud Hospital HF*

1. **Background**
   1. **General background**

Chronic heart failure (HF) is one of the most important public health problems in cardiovascular medicine. In the western world its incidence is ~2% of the general population, reaching even higher levels in the ageing population (~10%) [1]. Despite improved treatment, hospital admissions for HF continue to rise and accounts for ~5% of medical admissions in these countries [2] with high mortality ranging from 5-70% per year depending on the severity of the disease [1]. Chronic HF accounts for a considerable proportion of all cardiovascular related hospitalisations; about 20% of admissions and 30% of hospital days are due to this condition. The total economic burden amounts to 1-2% of total health care expenditure, of which hospitalisation costs make up two-thirds [3]. The most common cause of heart failure in the western developed countries is coronary artery disease, while cardiomyopathy is the most common in several developing countries. The exact contribution is, however not known, and depends on the age of the population and criteria used to determine the presence of heart failure. In the recent Euro Heart Failure survey, pure idiopathic cardiomyopathy was judged to account for 11% of the heart failure cases [4], while in a Norwegian database with 2550 cases 15% have dilated cardiomyopathy and 58% coronary artery disease, respectively (Morten Grundtvig, unpublished). In recent heart failure studies, coronary artery disease and non-ischemic cardiomyopathy have accounted for 54-71% and 29-46% of the cases, respectively [5]. The absence of significant coronary artery disease is a prerequisite for the diagnosis of idiopathic dilated cardiomyopathy (IDCM). IDCM represents the final common expression of primary myocardial damage produced by a variety of as yet undefined myocardial insults, producing areas of interstitial and perivascular fibrosis, particularly of the left ventricle.

Current evidence-based therapy of chronic HF caused by left ventricular (LV) systolic dysfunction is mainly based on inhibition of neurohumoral stimulation secondary to pump failure. ACE inhibitors and ß-blockers, in combination with diuretics are now first choice therapy [6-9]. Also spironolactone may be of benefit in advanced CHF [10]. Digitalis, hydralazine and long-acting nitrates do not affect neurohormones, but have other favourable effects.

**1.2. Inflammation in HF**

Despite state-of-the-art cardiovascular treatment, chronic HF is a progressive disease with high morbidity and mortality [11], suggesting that important pathogenic mechanisms remain active and unmodified by the present treatment modalities. The presence of chronic inflammation in patients with chronic heart failure has been widely recognized and coupled with persistent immune activation may represent such unmodified mechanisms. Thus, accumulating evidence indicates that inflammatory cytokines play a pathogenic role in chronic HF by influencing heart contractility, inducing hypertrophy, and promoting apoptosis or fibrosis, contributing to the continuous myocardial remodeling process [12-14]. While several stimuli may be operating such as heat shock protein, microbial antigens, shear and oxidative stress, hypoxia and oxidized-LDL, it seems that the inflammatory response to these stimuli may represent a common final pathogenic pathway in chronic HF regardless of the initial event and etiology of HF promoting progressive myocardial failure [13]. In accordance with this we have previously demonstrated that the immune response is similar between patients whose etiology are coronary artery disease or dilated cardiomyopathy [15]. We and others have suggested that this persistent immune activation and inflammation may represent “unmodified mechanisms” in chronic HF, and forms of immunomodulatory therapy in addition to conventional cardiovascular treatment regimens has emerged as possible new and promising treatment modalities in this disorder. However, except for small studies demonstrating a favorable effect of intravenous immunoglobulin [16] or pentoksifyllin, [17] other studies, especially anti TNFα therapy [18;19], have been disappointing, underscoring the need for additional studies in this research field.

**1.3. Anti-inflammatory effects of statins**

Several randomized trials, lowering cholesterols with HMG CoA reductase inhibitors; statins, have proven a reduction in morbidity and mortality due to atherosclerotic disease [20-22]. In addition, it has been shown that treatment of ischemic HF patients with a statin improves LVEF significantly [23]. However, recent studies suggest that the beneficial effects of statins also may be related to their anti-inflammatory properties. Thus, several *in vitro* studies and studies in animal models, as well as some studies in patients with cardiovascular disease, have shown the ability of statins to down-regulate levels of inflammatory cytokines, suppress inappropriate endothelial cell activation and to inhibit leukocyte-endothelial cell interactions. Furthermore, *in vitro* experiments and studies in animal models have shown that statins may prevent the development of cardiac hypertrophy in a cholesterol-independent manner involving immunomodulatory as well as anti-oxidant and matrix metalloproteinase’s (MMP)-inhibiting effects of these medications.[24] In fact, the combination of cholesterol-lowering, immunomodulatory and anti-oxidant properties suggest that statins should be an interesting therapeutic approach in HF regardless of etiology. However, the loss of the protection that lipoproteins may provide through binding and detoxifying endotoxins, such as lipopolysaccharide (LPS), entering the circulation via the gut may be potential harmful in HF patients.[25] Thus, in patients with HF it has been postulated that lower levels of LDL may be predictors of mortality [26]. IDCM patients seem to have lower levels of LDL compared with patients who have ischemic HF [26]. Vredevroe et al. [27]. have demonstrated that lower lipid levels predict mortality only in HF patients with IDCM and not in ischemic HF patients. Thus, there is a need for a definitive outcome trial to assess the efficacy and safety of statins in HF (both ischemic and IDCM), and two large studies in HF due to coronary artery disease (CAD) are ongoing (CORONA, GISSI).

**2. Rationale**

Based on the issues outlined above, statins should potentially not only be beneficial in HF secondary to CAD, but also in HF secondary to idiopathic dilated cardiomyopathy (IDCM).

- Approximately 35-45% of all HF patients have non-ischemic etiology of which many have pure IDCM
- IDCM is characterized by its lack of manifest vascular disease and hyperlipidemia
- HF patients, regardless of etiology have chronic inflammation
- Treating IDCM patients with a statin will lead to an attenuation of inflammatory marker levels as well as an amelioration of LVEF
- IDCM patients present a non-hyperlipidemic model in which we will be able to investigate and attempt to elucidate how modulating markers of inflammation with a potent statin may contribute to an amelioration of LVEF

To further elucidate this issue we want to study the potent new statin Rosuvastatin on myocardial function and remodeling and their relation to inflammatory markers in patients with IDCM. As hyperlipidemia is not involved in the pathogenesis of IDCM, as opposed to HF secondary to CAD, such studies will also be an interesting approach in separating the lipid lowering from other effects of these medications in HF.

**3. Objectives**

1. The primary objective of this study is to evaluate the effect of Rosuvastatin on LV remodeling in patients with HF secondary to IDCM. LV remodeling will be evaluated with magnetic resonance imaging (MRI) which offers an unsurpassed precision in the measurements of heart volumes and function. End points will be LV end systolic and diastolic volume (LVESV, LVEDV), regional wall motion score index (WMSI) and LV-ejection fraction (LV-EF).
2. The secondary objective of this study is to evaluate the effect of Rosuvastatin on:
   1. Effect on immunological variables
      1. Inflammatory cytokines: e.g., TNF-α, sTNFR2, interleukin-6 (IL-6).
      2. Anti-inflammatory cytokines: e.g., IL-10
      3. Chemokines: e.g., monocyte chemoattractant peptide-1 (MCP-1) and IL-8
      4. Regulators of matrix degradation: e.g., MMPs and their endogenous inhibitors TIMPs.
   2. the B-type natriuretic peptide (BNP), a sensitive marker of the degree of myocardial failure besides being a prognostic indicator in HF patients [28-30].
   3. Quality of life
   4. Effect on New York Heart Association (NYHA) functional class
   5. Effect on hospitalization and heart transplantation
   6. Effect on neurohormones
   7. Withdrawals
   8. Side effects

**4. Materials and methods**

**4.1. Patients**

*Inclusion*

Altogether 75 patients ill be enrolled. The criteria for enrollment in the study are clinical evidence of heart failure despite the conventional therapy and a left ventricular ejection fraction below 40%. The patients should have IDCM . Ischemic and primary valvular heart disease should be excluded by history, angiography or echocardiography.

Inclusion criteria

1. Age of 18-80 years
2. Have clinical or symptomatic evidence of HF, in NYHA class II-IV, for at least 3 months
3. Have LVEF <40%,
4. On optimal medical treatment and considered unsuitable for surgical intervention.
5. Have given written informed consent
6. No planned heart transplantation
7. Female of potential childbearing age must have a negative serum pregnancy test within 7 days prior to enrollment. Effective contraception must be used during the trial and for 6 weeks following discontinuation of the study medication, even where there has been a history of infertility.

*Exclusion criteria:*

1. Evidence of unstable disease
2. Evidence of ischemic etiology on the basis of history (diagnosed myocardial infarction), echocardiography or angiography)
3. Evidence of clinical significant valvular disease based on echocardiography
4. Significant concomitant diseases such as infections, pulmonary disease or connective tissue disease.
5. Contraindication against statin therapy

- hypersensitivity against statins
- liver disease with SGOT and SGPT > 2 timer upper normal limit
- Baseline elevations of CK 3 x ULN at any time during the course of the study
- Serum creatinine 2.0 mg/dL (177 umol/L) at any time during the course of the study
- pregnancy or breast feeding
- fertile women not using a contraceptive
- Cyclosporin therapy

1. Other illnesses or treatments which reduce the safety and/or efficacy of the treatment

- Hemodynamic important valvular disease requiring valvular replacement
- Established statin treatment
- Gastrointestinal treatment with possible malabsorption of the drug
- Cancer
- Serious psychiatric disease
- Life threatening ventricular arrhythmias
- Other medication increasing the risk of rhabdomyolysis

1. Participating in other studies

**4.2.** **Study design**

Patients who satisfy the inclusion and exclusion criteria will be randomized in a 1:1 ratio to Rosuvastatin or placebo. Rosuvastatin will be given in a daily dose of 10 mg. Duration of therapy will be of minimum 6 months, with an extra follow-up after 12 months.

##### 4.3 Dosing.

In the present study Rosuvastatin will be given in a dose of 10 mg daily. At the moment there is no consensus on the proper dosing of statins in HF. In general the concept of the lower (of LDL cholesterol) the better is getting acceptance for treatment of patients with coronary artery disease, but whether this also holds true in the treatment of HF is unknown. In the ongoing CORONA study, Rosuvastatin is given at a dose of 10 mg daily. We will therefore give the same dose in the present study. Specific reasons for discontinuing a subject from study medication are:

1. Withdrawal of informed consent

2. If, at any time, the subject’s CK measures >10 x ULN and is accompanied by

unexplained muscle pain, tenderness or weakness

3. If persistent ALT levels >3 x ULN on two occasions at least 48 hours apart are

demonstrated

4. If there is deterioration in the subject’s condition which, in the opinion of the

investigator, warrants study medication withdrawal

5. If there is the occurrence of an adverse event which, in the opinion of the

investigator, warrants study medication withdrawal

6. At the investigator’s discretion

##### 4.4 Measurements.

**4.4.1 Measurement of LV remodeling**

**MR**

This is performed by using a Siemens 1.5 tesla scanner. Standard MR recording of the left ventricle is done before injection of Gadolinium, 5 min after and between 10-20 min after the injection. This procedure shows areas of fibrosis and inflammation. In addition recordings for MR tagging are done.

Recordings are done before start of treatment and at the end point. EF, systolic and diastolic volume are measured. Areas of fibrosis and inflammation are described. Longitudinal, circumferential and radial strains in the different segments are measured and rotation quantified.

**Echocardiography**

A GE VIVID 7 ultrasonic digital device is used. The patients are examined in the lateral recumbent position after 15 min of rest. The heart is visualized by the standard ultrasonic techniques and acoustic windows giving a total homodynamic and valvular assessment. In addition tissue Doppler examinations are performed from the three apical planes and from parasternal short axis at the level of mitral valve and papillary muscles. These are done by using color coding, and single pulsed Doppler at the lateral mitral ring. Short axis recordings are then performed for tissue tracking analysis. The levels are parasternal short axis mitral, at the top of the papillary muscles and distal in the apex region. The last recording is obtained by moving the probe towards apex and laterally to get perpendicular scanning of the region. The speckle tracking recordings must have good echoes from the wall, i.e. the frequency must often be at 1.7 MHz, but can be increased towards 2.0. The focus must be localized in mid cavity level. Frame rate must be at the level of 60-80 Hz. The whole sector must be filled by the left ventricle. The same requirements are needed for the standard apical tissue recordings so that these can be used for radial strain measurements by speckle tracking technique.

Three heart beats are needed for this analysis. Recordings are performed before treatment and at end point. Analysis is done blinded when the patients have come to the end point. Standard measurements are done. In addition, strain and strain rate measurements are done in the different segments in longitudinal and radial direction. Rotation is measured from the speckle tracking recordings.

**4.4.2 Measurements of N-terminal proBNP (NT-proBNP)**

NT-proBNP will be measured by radioimmunoassay (Elecsys; Roche Diagnostics, Indianapolis, IN).

**4.4.3 Routine biochemical analysis and measurements of immunological variables**

Blood samples will be taken at baseline, after 2, 4 weeks, and 3 and 6 months for routine tests and inflammatory variables (baseline, 4 weeks, 3 and 6 months). Cytokines and other immunologic variable will be analysed in plasma/serum and in whole blood and peripheral blood mononuclear cells by enzyme immunoassays, bioassays, flow cytometry, immunohistochemistry, real-time RT-PCR, RNase protection assay and cDNA microarray.

##### 4.5 End points and statistics

Appropriate statistical analysis will be performed. The primary end points are changes in LV-EF as measured by MR. In order to observe an increase of LV-EF of 5% during Rosuvastatin compared to placebo at the end of the study with an α of 5% and power of 80% we will need approximately 32 patients in each group based on a standard deviation of LV-EF measurement of 7.5%. To compensate for possible drop out, and increase the chance of significant differences in secondary and tertiary end-points, 75 patients will be included.

## 5. Safety

5.1 Adverse events

An adverse event is the appearance or worsening of any undesirable sign, symptom, or medical condition occurring after starting the study drug even if the event is not considered to be related to study drug. Study drug includes the investigational drug under evaluation and the comparator drug or placebo that is given during any phase of the trial. Medical conditions/diseases present before starting study drug are only considered adverse events if they worsen after starting study drug. Abnormal laboratory values or test results constitute adverse events only if they induce clinical signs or symptoms, are considered clinically significant, or require therapy.

The occurrence of adverse events should be sought by non-directive questioning of the patient at each visit during the study. Adverse events also may be detected when they are volunteered by the patient during or between visits or through physical examination, laboratory test, or other assessments. As far as possible, each adverse event should be evaluated to determine:

1. the severity grade (mild, moderate, severe)
2. its relationship to the study drug(s) (suspected/not suspected) its duration (start and end dates or if continuing at final exam)
3. action taken (no action taken; temporarily interrupted; study drug permanently discontinued due to this adverse event; concomitant medication taken; non-drug therapy given; hospitalization/prolonged hospitalization)
4. whether it is serious, where a serious adverse event (SAE) is defined as one which:

- is fatal or life-threatening
- results in persistent or significant disability/incapacity
- constitutes a congenital anomaly/birth defect
- requires inpatient hospitalization or prolongation of existing hospitalization, unless hospitalization is for:
- routine treatment or monitoring of the studied indication, not associated with any deterioration in condition (specify what this includes)
- elective or pre-planned treatment for a pre-existing condition that is unrelated to the indication under study and has not worsened since the start of study drug
- treatment on an emergency outpatient basis for an event not fulfilling any of the definitions of a SAE given above and not resulting in hospital admission
- is medically significant, i.e., defined as an event that jeopardizes the patient or may require medical or surgical intervention to prevent one of the outcomes listed above.

**5.2 SAE’s occurring after Patient Randomization**.

Any serious adverse event occurring after the patient has been randomized and until 4 weeks after the patient has stopped study must be reported. This includes the period in which the study protocol interferes with the standard medical treatment given to a patient (e.g. change in treatment to a fixed dose of concomitant medication).

## 5.3 Adverse event reporting

### Serious Adverse event reporting by the Investigator:

The Investigator will delegate the electronic reporting of individual case safety reports to AstraZeneca AS, who will forward the report (E2B form) to NoMA.  In order to keep timelines, Investigator must forward copies of all SAEs to AstraZeneca within 1 day (ie, immediately but no later than the end of the next business day) of when he becomes aware of it. AstraZeneca AS will forward the quarterly line listings of suspected adverse reactions that are serious and unexpected to NoMA.

Recurrent episodes, complications, or progression of the initial SAE must be reported as follow-up to the original episode within 24 hours of the investigator receiving the follow-up information. An SAE occurring at a different time interval or otherwise considered completely unrelated to a previously reported one should be reported separately as a new event.

Information about all SAEs is collected and recorded on the Serious Adverse Event Report Form. The original copy of the SAE Report Form and the fax confirmation sheet must be kept with the case report form documentation at the study site.

Follow-up information is sent to the same person to whom the original SAE Report Form was sent, using a new SAE Report Form stating that this is a follow-up to the previously reported SAE and giving the date of the original report. Each re-occurrence, complication, or progression of the original event should be reported as a follow-up to that event regardless of when it occurs. The follow-up information should describe whether the event has resolved or continues, if and how it was treated, whether the blind was broken or not, and whether the patient continued or withdrew from study participation.

## 6. Treatment blinding

This is a double-blind study. Since Rosuvastatin is expected to lower cholesterol, investigators must agree on not analyzing cholesterol levels in their subjects during the double blind part of the study unless judged absolutely necessary by the investigator in order to obtain blindness.

**7. Informed consent**

Informed consent will be documented by the dated signature of the patient, or by the signature of the patient’s legal guardian. The signature will confirm that the consent has been given following both oral and written information, which has been understood. The investigator will keep the signed informed consent forms and is available for inspection by the authorities. The participating centres will use local guidelines for the oral information.

### 8. Ethical considerations

Chronic HF is a debilitating disease for which there are no effective treatments. The use of statin in previous clinical trials has indicated potential of the drug composition for effective treatment of CHF. The patients will be fully informed, both orally and in writing, of the purpose and the rationale for the trial as well as the additional tests, which are part of the trial. It is thus considered ethically acceptable to test the efficacy and possible side effects of statin treatment in patients with IDCM. The study will be forwarded to the Regional Ethical committee and to Statens Legemiddelverk in Norway

.

### 9. Trial chart

The patients will be seen and evaluated according to the study evaluation schedule below*.*

|  | Pre | Rand | 4 w | 3 mo w | 6 mo | 12 mo |
| --- | --- | --- | --- | --- | --- | --- |
| Informed consent^1^ | X |  |  |  |  |  |
| Pregnancy test ^2^ | X |  |  |  |  |  |
| Medical history | X |  |  |  |  |  |
| Physical examination | X | X | x | x | X | X |
| NYHA | X | X | x | x | X | X |
| MRI^3^ |  | X |  |  | X |  |
| Routine Lab tests^4^ | X |  | x | x | X | X |
| BNP/Cytokines^5^ |  | X |  | x | X | X |
| Tolerability^6^ |  |  | x | x | X |  |
| Compliance^7^ |  |  | x | x | X |  |
| Adverse and clinical events |  | X | x | x | X |  |
| Dispense study drug^8^ |  | x |  | X |  |  |
| Quality of life^9^ |  | x |  |  | X |  |
| Overall treatment evaluation^10^ |  |  |  |  | X |  |
| Gene expression ^11^ |  | X |  |  | X | x |

**Legend:**

1. The informed consent must be signed **prior to** the baseline assessments and the study entry.

2. Only females of potential childbearing. Test must be available and negative prior to randomization.

3. MRI is performed at Rikshospitalet-Radiumhospitalet or Ullevål University Hospital.

4. Routine Biochemistry (Lab tests) Hb, white count, platelets, sodium, potassium Kreatinin, urea, CK, total-cholesterol, LDL-cholesterol, HDL-cholesterol and triglycerides. Can be done either at screening or at baseline

5. BNP and Cytokines see 4.4.3 and 3.2.a

6. Tolerability for study drugs will be asked at each visit. Tolerability will be evaluated as part of adverse events analyses

7. SAEs, infections and pregnancies should be reported for up to 30 days after the last dose of study medication.

8. First dose of study drug has to be started the same day as randomization

9. Quality of life will be measured by the Minnesota Living with Heart Failure questionnaire and EuroQol questionnaire

10 Overall treatment evaluation is evaluated by a separate questionnaire

11. Gene expression is done by RT-PCR of RNA extracted from white blood cells.

**10. Data review and database management**

A separate CRF will be used (see attachment). The investigator must maintain source documents for each patient in the study, consisting of case and visit notes (hospital or clinic medical records) containing demographic and medical information, laboratory data, electrocardiograms, and the results of any other tests or assessments. All information on CRFs must be traceable to these source documents in the patient's file. Data not requiring a written or electronic record will be defined before study start and will be recorded directly on the CRFs, which will be documented as being the source data. The investigator must also keep a copy of the signed informed consent form.

One of the study personnel will checks of the consistency of the source data with the eCRFs.

The tablets will be packed and dispensed by the pharmacy at Rikshospitalet-Radiumhospitalet, who will keep the randomization code

## 11. Treatments (study drug, concomitant therapies, compliance)

Duration (days) of study medication administration will be summarized; this will include periods of temporary interruption of study medication for safety reasons. Further, frequency of dose reduction (including temporary dose interruption) for safety reasons as per protocol guidelines as well as average daily dose will be presented. In calculating dosage averages, zero doses will be used for periods of temporary interruption of study medication regardless of whether this was due to safety reasons or patients’ non-compliance.

Concomitant medication will be coded according to the ATC (Anatomical, Therapeutic, Chemical) classification system, and will be tabulated descriptively.

**12. References**

1. Cowie MR, Mosterd A, Wood DA, et al. The epidemiology of heart failure. Eur Heart J 1997;18:208-225

2. Stewart S, MacIntyre K, MacLeod MMC, Bailey AM, Capwell S, McMurray JJ. Trends in hospitalization for heart failure in Scotland, 1990-1996. Eur Heart J 2001;22:209-217

3. McMurray J, Hart W, Rhodes G. An evaluation of the cost of heart failure to the National Health Service in the UK. Br J Med Econ 1993;6:99-110

4. Cleland JG, Swedberg K, Follath F, et al. The EuroHeart Failure survey programme-- a survey on the quality of care among patients with heart failure in Europe. Part 1: patient characteristics and diagnosis. European Heart Journal 24(5):442-63, 2003;

5. McMurray JJ, Stewart S. Epidemiology, aetiology, and prognosis of heart failure. Lancet 2000;83:596-602

6. The CONSENSUS Trial Study Group. Effects of enalapril on mortality in severe congestive heart failure: Results of the Cooperative North Scandinavian Enalapril Survival Study (CONSENSUS). N Engl J Med 1987;316:1429-1435

7. The SOLVD Investigators. Effect of enalapril on mortality and the development of heart failure in asymptomatic patients with reduced left ventricular ejection fraction. N Engl J Med 1992;327:685-691

8. Packer M, Bristow MR, Cohn JN, et al. The effect of carvedilol on morbidity and mortality in patients with chronic heart failure. N Engl J Med 1996;334:1349-1355

9. MERIT-HF Study Group. Effect of metoprolol CR/XL in chronic heart failure: Metoprolol CR/XL randomised intervention trial in congestive heart failure. Lancet 1999;353:2001-2007

10. Pitt B, Zannad F, Remme WJ, et al. The effect of spironolactone on morbidity and mortality in patients with severe heart failure. N Engl J Med 1999;341:709-717

11. Doughty RN, Whalley GA, Walsh HA, Gamble GD, Lopez-Sendon J, Sharpe N. Effects of carvedilol on left ventricular remodeling after acute myocardial infarction. The CAPRICORN echo substudy. Circulation 2004;109:201-206

12. Sasayama S, Matsumori A, Kihara Y. New insights into the pathophysiological role for cytokines in heart failure. Card Res 1999;42:557-564

13. Mann DL. Inflammatory mediators and the failing heart. Circ Res 2002;91:988-998

14. Damås JK, Gullestad L, Aukrust P. Cytokines as new treatment for heart failure. Curr Control Trials Cardiovasc Med 2001;2:271-277

15. Aukrust P, Ueland T, Lien E, et al. The cytokine network in congestive heart failure-dysbalance between pro-inflammatory and anti-inflammatory mediators. European Heart Journal 19, 70 (abstract). 1998.
Ref Type: Abstract

16. Gullestad L, Aass H, Fjeld JG, et al. Effect of immunomodulating therapy with intravenous immunglobulin in chronic congestive heart failure. Circulation 2001;103:220-225

17. Skudicky D, Bergemann A, Sliwa K, Candy G, Sareli P. Beneficial effects of pentoxifylline in patients with idiopathic dilated cardiomyopathy treated with angiotensin-converting enzyme inhibitors and carvedilol. Circulation 2001;103:1083-1088

18. Louis A, Cleland JGF, Crabbe S, et al. Clincal trial update. Eur J Heart Fail 2002;3:381-387

19. Lisman KA, Stetson SJ, Koerner T, et al. The role of tumor necrosis factor alpha in the treatment of congestive heart failure. Congest Heart Failure 2002;8:275-279

20. Scandinavian Simvastatin Survival Study Group. Randomized trial of cholesterol lowering in 4444 patients with coronary artery disease: the Scandinavian Simvastatin Survival Study (4S). Lancet 1995;344:1383-1389

21. The LIPID study Group. Prevention of cardiovascular events and death with pravastatin in patients with coronary artery disease and broad range of initial cholesterol levels. New Engl J Med 1998;339:1349-1357

22. Sacks FM, Pfeffer MA, Moyle LA, et al. The effect of pravastatin on coronary events after myocardial infarction in patients with average cholesterol levels. N Engl J Med 1996;335:1001-1009

23. Hong YJ, Jeong MH, Hyun DW, et al. Prognostic significance of simvastatin therapy in patients with ischemic heart failure who underwent percutaneous coronary intervention for acute myocardial infarction. American Journal of Cardiology 95(5):619-22, 2005;

24. Takemoto M, Node K, Nakagami H, et al. Statins as antioxidant therapy for preventing cardiac myocyte hypertrophy. J Clin Invest 2001;108:1429-1437

25. Krum H, McMurray JJ. Statins and chronic heart failure: do we need a large scale outcome trial? J Am Coll Cardiol 2002;39:1567-1573

26. Conraads VM, Bosmans JM, Schuerwegh AJ, et al. Association of lipoproteins with cytokines and cytokine receptors in heart failure patients. Differences between ischaemic versus idiopathic cardiomyopathy. European Heart Journal 24(24):2221-6, 2003;

27. Vredevoe DL, Woo MA, Doering LV, Brecht ML, Hamilton MA, Fonarow GC. Skin test anergy in advanced heart failure secondary to either ischemic or idiopathic dilated cardiomyopathy. American Journal of Cardiology 82(3):323-8, 1998;

28. Maise.A. B-type natriuretic peptide levels: diagnostic and prognostic in congestive heart failure. Circulation 2002;105:2328-2331

29. Berger R, Huelsman M, Strecker K, et al. B-type natriuretic peptide prdicts sudden dath in patients with chronic heart failure. Circulation 2002;105:2392-2397

30. Cheng V, Kazanagra R, Garcia A, Lenert LKPGN, Clopton P, Maisel A. A rapid bedside test for B-type peptide predicts treatment outcomes in patients admitted for decompensated heart failure. J Am Coll Cardiol 2001;37:386-391

31. Wæhre T, Yndestad A, Smith CR, et al. Increased expression of interleukin-1 in coronary artery disease and downregulatory effects og HMG-CoA reductase inhibitors. Circulation 2004;109:1966-1972

32. Wæhre T, Damås JK, Gullestad L, et al. Hydroxymethylglutaryl coenzyme A reductase inhibitors down-regulate chemokines and chemokine receptors in patients with coronary artery disease. The Journal of American College of Cardiology 41, 1460-1467. 2003.
